# Fluorescence based HTS-compatible ligand binding assays for dopamine D_3_ receptors in baculovirus preparations and live cells

**DOI:** 10.3389/fmolb.2023.1119157

**Published:** 2023-03-16

**Authors:** Maris-Johanna Tahk, Tõnis Laasfeld, Elo Meriste, Jose Brea, Maria Isabel Loza, Maria Majellaro, Marialessandra Contino, Eddy Sotelo, Ago Rinken

**Affiliations:** ^1^ Institute of Chemistry, University of Tartu, Tartu, Estonia; ^2^ Department of Computer Science, University of Tartu, Tartu, Estonia; ^3^ Centro Singular de Investigación en Medicina Molecular y Enfermedades Crónicas (CiMUS), Universidade de Santiago de Compostela, Santiago, Spain; ^4^ Centro Singular de Investigación en Química Biolóxica e Materiais Moleculares (CiQUS), Universidade de Santiago de Compostela, Santiago, Spain; ^5^ Celtarys Research S.L., Santiago, Spain; ^6^ Dipartimento di Farmacia-Scienze del Farmaco, Università degli Studi di Bari Aldo Moro, Bari, Italy

**Keywords:** G protein-coupled receptor (GPCR), dopamine D3 receptor (D3R), ligand binding kinetics, fluorescent probe, deep-learning based image analysis, budded baculoviruses, fluorescent microscopy, fluorescence polarization (FP)

## Abstract

Dopamine receptors are G-protein-coupled receptors that are connected to severe neurological disorders. The development of new ligands targeting these receptors enables gaining a deeper insight into the receptor functioning, including binding mechanisms, kinetics and oligomerization. Novel fluorescent probes allow the development of more efficient, cheaper, reliable and scalable high-throughput screening systems, which speeds up the drug development process. In this study, we used a novel Cy3B labelled commercially available fluorescent ligand CELT-419 for developing dopamine D3 receptor-ligand binding assays with fluorescence polarization and quantitative live cell epifluorescence microscopy. The fluorescence anisotropy assay using 384-well plates achieved Z’ value of 0.71, which is suitable for high-throughput screening of ligand binding. The assay can also be used to determine the kinetics of both the fluorescent ligand as well as some reference unlabeled ligands. Furthermore, CELT-419 was also used with live HEK293-D3R cells in epifluorescence microscopy imaging for deep-learning-based ligand binding quantification. This makes CELT-419 quite a universal fluorescence probe which has the potential to be also used in more advanced microscopy techniques resulting in more comparable studies.

## 1 Introduction

Dopamine receptors are G-protein-coupled receptors (GPCRs), which have five subtypes - D_1-5_. The dysfunction of these receptors has been linked to the development of many serious pathologies, like depression, schizophrenia and Parkinson’s disease (Leggio et al., 2016; Sokoloff and Le Foll, 2017). Therefore, numerous dopamine ligands have been approved as drugs. However, these drugs often have serious side effects and too low or temporary therapeutic effects (Borovac, 2016; Garcia-Borreguero et al., 2016). As dopaminergic signalling is quite complex, there is a need for novel assays to study this system. Ligand binding affinity along with the kinetic properties determine if a ligand is a suitable drug candidate. Although kinetic parameters are difficult to obtain with endpoint assays, several luminescence-based (Borgarelli et al., 2021), but also label-free methods (Rascol et al., 2021), which allow online monitoring of ligand binding, have emerged as promising alternatives.

Even though the first fluorescent ligands for dopamine D_1_ and D_2_ receptors were already published in 1989 (Monsma Jr. et al., 1989; Barton et al., 1991), they have not found wide usage in binding assays so far. Since then, several next-generation fluorescent ligands have been developed for different dopamine receptor subtypes, including D_3_ receptors, which have found use in multiple assays. For example, in 2015, D_3_ receptor partial agonist BP 897 was labelled with either DY647 or fluorescein and used in a time-resolved fluorescence resonance energy transfer assay to study D_1_/D_3_ receptor hetero-oligomers (Hounsou et al., 2015). In 2016 several Cy3B labelled ligands were synthesized to study dopamine receptor dimerization with TIRF microscopy (Tabor et al., 2016). That set of Cy3B labelled fluorescence ligands was very diverse, including agonists based on (S)-5-OH-DPAT and antagonists as well as bivalent ligands both based on 1,4-disubstituted phenylpiperazine. In a more recent study, the indanylamine pharmacophore was labelled with Alexa488, Cy3B, and tetramethylrhodamine dyes (TAMRA) to be used in different assays (Allikalt et al., 2020). All three ligands were tested in the nanoBRET assay, where the affinities obtained were similar to the sub-nanomolar affinities obtained from the radioligand binding assay. Furthermore, to visualize D_2_ and D_3_ receptors in TIRF microscopy, the Cy3B-labeled probe was used.  Moreover, NAPS-Cy3B was used to develop a quantitative live cell microscopy assay (Allikalt et al., 2021). This assay allows high-content ligand binding studies in live cells by utilizing a machine-learning model for cell contour detection from bright-field microscopy images and fluorescence channel for binding quantification. The most recent development in fluorescence methods to study D_3_ receptors is the PharmacoSTORM utilizing Sulfo-Cy5-labeled cariprazine to quantify drug-target interaction sites at the nanoscale level within complex tissue preparations (Prokop et al., 2021). The problem with all these ligands is their unavailability, as they all are synthesized in-house for a particular study and are not commercially available for either research or HTS applications. However, commercial alternatives also exist, for example, D_1_-like receptor ligands from Hello Bio and D_2_-like receptor ligands from Celtarys Research.

A better understanding of ligand binding kinetics is necessary for a more mechanistic and systematic insight into drug action. Therefore, fluorescence polarization assays and other fluorescence based methods allowing kinetic measurements help reduce this fundamental knowledge gap. Fluorescence anisotropy (FA) assay has been used to study the ligand binding kinetics of many GPCRs - 5-HT_1A_ (Tõntson et al., 2014), melanocortin MC_4_ (Link et al., 2017), dopamine D_1_ (Allikalt et al., 2018), neuropeptide Y Y_1_ (Müller et al., 2022), muscarinic acetylcholine M_2_ (Grätz et al., 2021) and M_4_ (Tahk et al., 2022) receptors. FA is based on measuring the change in rotational freedom of the fluorescent label upon receptor binding and, therefore, does not require the physical separation of the bound and free ligand (Rinken et al., 2018). Upon receptor-ligand complex formation, the rotational diffusion time of the bound ligand should be significantly hindered compared to the fluorescence lifetime of the label to obtain a large change in FA. Moreover, due to the ratiometric nature of the assay, the FA signal depends on the concentration of both the free and the receptor-bound fluorescent ligand. Therefore, FA requires sufficient receptor concentration to achieve free achieve depletion of free fluorescent ligand. Budded baculoviruses (BBV) have a high receptor concentration as well as being homogenous in size, which makes them a good receptor source for FA assay. (Rinken et al., 2018). As the kinetic mechanisms of ligand binding can be more complex compared to equilibrium models and ligand depletion requirement of FA assay leads to second-order kinetic conditions it is necessary to apply appropriate data analysis techniques to such data. A suitable solution is provided by global analysis techniques using systems of ordinary differential equations which are typically implemented in systems biology software (Beechem et al., 2002). Global analysis with systems biology software typically allows avoiding multiple problems such as model-imposed restrictions to experimental design, more efficient search and validation of the correct binding mechanism and in this case accurate prediction of kinetic parameters of both labeled and unlabeled ligands.

It must be considered that the BBVs are nanoparticles covered with Sf9 cell membranes that have a different membrane composition compared to mammalian cells (Marheineke et al., 1998) which also may affect receptors’ properties (Harikumar et al., 2005; Mondal et al., 2014). BBVs also lack downstream signalling components like G-proteins and β-arrestins, which modulate the ligand binding affinities and receptor trafficking. The more straightforward system allows for studying ligand binding without additional interference and sources of variability, but it does not allow for the investigation of the full complexity of the system. Therefore, to obtain a more complete picture, it is important to measure ligand binding in a more natural system like live mammalian cells. As mentioned before, quantitative live cell microscopy assay (Allikalt et al., 2021) has been developed for D3 receptors, and further improvements were made with the muscarinic acetylcholine M_4_ receptor. This method relies on cell segmentation from the bright-field channel while quantifying ligand binding from the fluorescence channel. The advantage of this approach is that no additional fluorescence channels are needed for cell and cell contour detection reducing both photo and chemical toxicity while also allowing for the analysis of different areas of the cell depending on the cell type or the goal of the analysis. The method has been further developed to allow for kinetic measurements and reduced data volumes by employing deep convolutional neural networks, which regularly surpass more classical machine learning models such as random forests (Caicedo et al., 2019; Tahk et al., 2022). Nowadays, many machine learning models can be developed in almost any available high-performance cluster or even on a reasonably powerful PC, but it is also possible to use freely available open-source cloud-based tools for the task. Generally, preferring these open-source community-based tools is likely to lead to better and more robust models even if the user has not received extensive training in computer science as tutorials and explanations are provided which helps to avoid common pitfalls. Some of such tools also provide pre-trained machine learning models which could work for a specific assay without any need for retraining a model. In the present study, the open-source ZeroCostDL4Mic framework was used for developing the deep convolutional neural network cell segmentation model with the corresponding Jupyter notebooks (Kluyver et al., 2016).

To cross-validate the developed assays, we employed three complementary methods – radioligand binding, FA and live-cell microscopy assay to measure the binding affinity of CELT-419 to D_3_ receptors. The possibility to use CELT-419 in different types of assays allows high flexibility of further assay development towards fully automated high-throughput screening of unlabeled ligands or more advanced live-cell microscopy-based assays.

## 2 Materials and methods

### 2.1 Materials

The assay buffer consisted of MilliQ water, 11 mM Na-HEPES (pH = 7.4) (Sigma-Aldrich), 135 mM NaCl (AppliChem), 1 mM CaCl_2_ (AppliChem), 5 mM KCl (AppliChem), 1 mM MgCl_2_ (AppliChem), protease inhibitor cocktail (according to the manufacturer’s description, Roche) and 0.1% Pluronic^®^ F-127 (Sigma-Aldrich). Dithiothreitol (DTT) (AppliChem) was added to the assay buffer for experiments with dopamine and apomorphine with an end concentration of 1 mM.

D_3_ receptor ligands 7-hydroxy-DPAT hydrobromide were purchased from TOCRIS and Spiperone (Sigma S7395), Haloperidol (Sigma H1512), Dopamine, Apomorphine, and Butaclamol were from Sigma-Aldrich. The fluorescent ligand CELT-419, its pharmacophore P-165 and pharmacophore with linker PL-384 were kindly provided by Celtarys Research. Stock solutions of these ligands were prepared in DMSO (Applichem) or Milli-Q water in the case of Dopamine.

### 2.2 Cell culture

Spodoptera frugiperda Sf9 (Invitrogen) cells were cultured in serum-free Excell420™ (Sigma-Aldrich) and Mimic™ Sf9 Insect Cells were cultured in Excell420™ supplemented with 2% fetal bovine serum (Sigma-Aldrich) at 27 °C as a suspension culture.

Human embryonic kidney cell line HEK293 stably expressing human wild-type dopamine D_3_ receptors (HEK293-D3R) was generated by Dr Reet Reinart-Okugbeni as described in (Reinart-Okugbeni et al., 2012). The cells were grown as an adherent monolayer on BioLite Petri dishes (Thermo Fisher Scientific) in DMEM high glucose medium (Sigma-Aldrich) supplemented with 9% fetal bovine serum, antibiotic antimycotic solution (100 U/ml penicillin, 0.1 mg/ml streptomycin, 0.25 μg/ml amphotericin B) (Sigma-Aldrich), and 400 μg/ml geneticin (Capricorn Scientific) at 37°C in a humidified incubator with 5% CO_2_.

The density and viability of cells were determined with the addition of 0.2% trypan blue (Sigma-Aldrich) using an Automated Cell Counter TC20™ (Bio-Rad Laboratories, Sundyberg, Sweden).

### 2.3 Radioligand binding assay

All dopamine receptor competition binding experiments were carried out in a polypropylene 96-well plate. For D_3_ receptors in each well was incubated 2 μg of membranes from D_3_ cell line (Perkin Elmer, ES-173M400UA, protein concentration = 1000 μg/ml), 1 nM [^3^H]-Spiperone (68 Ci/mmol, 1 mCi/ml, Perkin Elmer NET1187001MC) with compounds studied or a standard. Non-specific binding was determined in the presence of 1 μM Haloperidol (Sigma H1512). The reaction mixture (V_total_: 250 μL/well) was incubated at 25 °C for 60 min, 200 μL was transferred to GF/C 96-well plate (Millipore, Madrid, Spain) pretreated with 0.5% of PEI and treated with binding buffer (50 mM Tris-HCl, 5 mM MgCl_2_, pH = 7.4), after which it was filtered and washed four times with 250 μL of wash buffer (50 mM Tris-HCl, pH = 7.4), before measuring in a microplate beta scintillation counter (Microbeta Trilux, PerkinElmer, Madrid, Spain).

For D_2_ receptors, in each well, 20 μg of membranes from CHO-D2#S20 cell line (Lot: A005/04-06-2020, protein concentration = 4322 μg/ml) were incubated, along with 1.5 nM [^3^H]-Spiperone (54.3 Ci/mmol, 1 mCi/ml, Perkin Elmer NET1187250UC), the compounds to be studied or the compound used as a standard. Non-specific binding was determined in the presence of 10 μM Sulpiride (S8010, Sigma Aldrich). The reaction mixture (V_total_: 250 μL/well) was incubated at 25 °C for 120 min. The following step are the same as for D_3_ receptors.

For Dopamine D_4_ receptor in each well was incubated 8 μg of membranes from hD4 (Millipore HTS223M) (Lot: SC232632, protein concentration = 1000 μg/ml), 1 nM [^3^H]-Spiperone (54.2 Ci/mmol, 1 mCi/ml, Perkin Elmer NET1187001MC) with compounds studied and a standard. Non-specific binding was determined in the presence of 25 μM Haloperidol (Sigma H1512). The reaction mixture (V_total_: 250 μL/well) was incubated at 27 °C for 120 min. The following steps are the same as for D_3_ receptors.

### 2.4 Preparation of budded baculovirus particles

The human D_3_ receptor in pcDNA3.1+ was purchased from the cDNA Resource Center (www.cdna.org). Construction and production of BBV containing D_3_ receptors were performed as described in (Veiksina et al., 2021) with some modifications. For cloning D_3_ into the pFastBac vector XhoI and XbaI/NheI sites were used. To transform the bacmid into Sf9 cells, the transfection reagent ExGen 500 (Fermentas) was used according to the manufacturer’s protocol. After the baculovirus preparations were generated and collected, the amount of infectious viral particles per ml (IVP/ml) for all the baculoviruses was determined with the Image-based Cell Size Estimation (ICSE) assay described in (Laasfeld et al., 2017). All the steps were carried out with Sf9 cells, except for the final budded baculovirus particle production which was done with Mimic™ Sf9 Insect cells, without additional FBS.

### 2.5 Fluorescence anisotropy experiments

FA experiments were carried out on black flat bottom 384 well plates (Corning) with a final volume of 30 µL.

In saturation binding experiments 3 nM and 0.5 nM, a fluorescent ligand was used with two-fold dilutions of BBV. To determine non-specific binding 10 or 50 µM Spiperone was used and to start dissociation 333 µM Spiperone was added.

For competition binding experiments, the concentrations of CELT-419 were kept constant at 0.5 nM and the volume of BBV was also constant at 1 μL (C_final_ ≈ 0.67 nM). The competitive ligand dilutions were done on the measurement plate. Also, a point with no competitive ligand was included and for blank correction, a point with only BBV was included. Measurements were carried out with 5 min intervals for 5 h at 27 °C. In all cases, BBV was added as the last component to initiate the ligand binding process.

In all experiments, the fluorescence intensity values were blank corrected for BBV autofluorescence and light scattering by subtracting the respective parallel or perpendicular fluorescence intensity value of a blank well from the respective measurement well. The blank wells lacked all the ligands and only contained the same concentration of BBVs as the measurement well.

FA measurements were performed with a multi-mode plate reader Synergy NEO (Biotek), which is equipped with a polarizing 530 (25) nm excitation filter and 590 (35) nm emission filter allowing simultaneous parallelly and perpendicularly polarized fluorescence detection. All experiments were carried out in duplicates at least three separate times.

### 2.6 Live-cell fluorescence microscopy

For fine-tuning the U-Net cell segmentation model for HEK293 cells, the fluorescence ground-truth masks were generated as described in (Tahk et al., 2022). Briefly, HEK293-D3R cells were seeded with a density of 20,000 cells/well into a µ-Plate 96 well Black well plate (Ibidi, Gräfelfing, Germany) and incubated for 2.5 h. One mM DiI (Invitrogen, Eugene, Oregon, United States) in DMSO kept at -20°C was thawed and, to disrupt aggregates, kept in an ultrasound bath for 5 min. To stain the cell membranes, the cell medium was replaced with 200 µL/well of 2 µM DiI in DPBS with Ca^2+^ and Mg^2+^. The cells were incubated with the DiI solution for 30 min before the imaging was started. Cytation 5 cell imaging multi-mode plate reader (BioTek) using a 20× LUCPLFLN objective (Olympus) was used for cell imaging from both fluorescence RFP channels, using 531 (40) nm excitation and 593 (40) nm emission filter, and the bright-field channel. Imaging parameters used during image acquisition were for bright-field: LED intensity = 4, integration time = 110 m, camera gain = 24 and for RFP fluorescence channel: LED intensity = 1, integration time = 71 m, camera gain = 24. A total of 196 fields of view were captured.

For live-cell microscopy saturation experiments with CELT-419, HEK293-D3R cells were seeded to µ-Plate 96 well Black well plate with a density of 20,000 cells/well. The cells were incubated for 5 h after which the cell medium was removed, cells were washed once with warm cell culture medium and finally, the ligand solutions in the cell culture media were added. For total saturation binding, two-fold serial dilutions of CELT-419 starting with 4 nM concentration were prepared. Non-specific binding control wells were prepared in the same manner but with the addition of 10 µM unlabeled D_3_ receptor antagonist Spiperone. Imaging parameters used during image acquisition were for bright-field: LED intensity = 5, integration time = 67 m, camera gain = 24 and for RFP fluorescence channel: LED intensity = 5, integration time = 557 m, camera gain = 22. The cells were imaged in the montage mode (4 locations per well) with *Z*-stack (10 planes, 4 planes below and 5 planes above focus) to reduce the influence of imaging location-dependent variability.

For live-cell microscopy competition experiments with CELT-419, HEK293-D3R cells were seeded to µ-Plate 96 well Black well plate with a density of 40,000 cells/well if the cells were incubated for 5 h and 20,000 cells/well if the cells were incubated for 24 h. After the incubation the cell medium was replaced with the cell culture media containing ligands. Imaging parameters used during image acquisition were for bright-field: LED intensity = 5, integration time = 85 m, camera gain = 20 and for RFP fluorescence channel: LED intensity = 2, integration time = 557 m, camera gain = 22. The cells were imaged in the montage mode (4 locations per well) with *Z*-stack (6 planes, 3 planes below and 2 planes above focus) to reduce the influence of imaging location-dependent variability.

### 2.7 Deep learning model development

The deep convolutional neural network was developed using the ZeroCostDL4Mic framework and Jupyter notebooks. These open-source frameworks are essentially reprogrammable data analysis reports, containing both text and figures but also include the python code used for the report generation. The possibility to document the code with text, figures and graphical user interface components means that using Jupyter notebooks does not necessarily require programming. The ZeroCostDL4Mic framework provides a number of predefined models, a wiki and video tutorials to help any researcher get accustomed to machine learning models and reduce the aforementioned problems with model development. For cell detection from bright-field images and quantification of fluorescence images, the *MembraneTools* toolbox of Aparecium software (https://www.gpcr.ut.ee/aparecium.html and https://github.com/laasfeld/Aparecium) was used, which is an open-source option for image and experimental data analysis and management software. Aparecium allows to easily analyze, store and document both endpoint and kinetic experiments.

Image analysis was performed as described in (Tahk et al., 2022) with some modifications. For training the final U-Net2D image segmentation model in the ZeroCostDL4Mic (Ronneberger et al., 2015; von Chamier et al., 2021) environment, U-Net2D notebook with minor modifications was used to allow compatibility with Aparecium and Matlab Keras framework model import. The modified Jupyter notebook is freely available at https://github.com/laasfeld/DL-For-HEK293. Model performance evaluation on an image test set gave F1 score of 0.76 and Matthew´s correlation coefficient of 0.75 calculated as described in (Tahk et al., 2022).

### 2.8 Data analysis

Aparecium 2.0 software (https://www.gpcr.ut.ee/aparecium.html and https://github.com/laasfeld/Aparecium) was used to blank the raw parallel and perpendicular intensity values and calculate the FA values using formula (1) (Jablonski, A, 1960).

$$FA\left(t\right)=\frac{{I\left(t\right)}_{II}-{I\left(t\right)}_{⊥}}{{I\left(t\right)}_{II}+2∙{I\left(t\right)}_{⊥}}$$ (1)

where *FA(t)* is the measured fluorescence anisotropy at time point *t* and *I(t)*_*II*_ and *I(t)*_⊥_ are parallel and perpendicular fluorescence intensities respectively at time point *t*.

To predict the fluorescence anisotropy value at any time point during kinetics experiments based on the instantaneous concentrations of each considered fluorescence ligand state in the systems biology analysis the following equation was used:

$$FA\left(t\right)=\frac{\sum {\left\{{Ligand\;binding\;state}_{i}\right\}}_{t}*FA\left({Ligand\;binding\;state}_{i}\right)}{\sum {\left\{{Ligand\;binding\;state}_{i}\right\}}_{t}}$$ (2)

Where *FA(t)* is the predicted fluorescence anisotropy at time point *t, {Ligand binding state*_*i*_ *}*_*t*_ represents the instantaneous concentration of fluorescence ligand in *i*th binding state at time point *t* and *FA*(*Ligand binding state*_*i*_) is the intrinsic fluorescence anisotropy value of fluorescence ligand in the *i*th binding state. Binding states of CELT-419 considered during fitting of equilibrium global analysis and systems biology analysis were free CELT-419, D_3_ receptor bound CELT-419 and non-specifically bound CELT-419.

The affinities (K_d_ of CELT-419 and K_i_ values of unlabeled compounds) from a certain timepoint were calculated from FA assays as described in (Veiksina et al., 2014) using GraphPad Prism 5.0 (GraphPad Software) and from radioligand assay using GraphPad Prism 7.00 with four-parameter logistic regression model and Cheng-Prusoff equation (Cheng and Prusoff, 1973).

To model the receptor-ligand binding system more precisely and correctly account for effects such as fluorescence ligand and non-labeled ligand depletion, non-specific binding perform fitting using global modeling a systems biology approach was taken. This allows system modeling with stronger constraints imposed by measurement data and fewer constraints imposed by artificial restrictions from assumptions used to obtain analytical equations. An ordinary differential equation system-based model was chosen where a single differential equation describes the concentration change of a single component. Systems biology analysis was performed for FA saturation binding experiments with BBV volumes lower than 1 µL/well (D_3_ receptor stock concentration corresponding to 20 ± 5 nM). Fitting was performed at two different CELT-419 concentrations which were measured as described in the saturation binding experiment description. Both the total and non-specific binding curves, as well as both the association and dissociation kinetic phases were used for fitting. For PL-384 association and dissociation kinetics determination, all the concentration points remaining on the slope of the concentration-response curve were used as well as one or two concentration points from both the upper and lower plateaus. Some concentrations from the upper and lower plateau were omitted as the plateau points contain relatively little information about competitive ligand kinetics, and omitting these points gave more weight to slope points to obtain more accurate k_on_ and k_off_ values for PL-384. Similarly, time scaling feature of IQMTools (“2: less timescaling”) was used to give more weight to the measurement points at the start of the measurement with more rapidly changing FA values Mean-squared error of the predicted FA value was used as the loss function. Using a global Nelder-Mead simplex-based simulated annealing algorithm (Nelder and Mead, 1965; Kirkpatrick et al., 1983) with starting temperature of 1000, iterations per temperature of 10^3^ and temperature reduction factor of 0.2, the temperature was reduced until it reached a value of 0.1. In the context of simulated annealing, the temperature refers to a parameter that controls how large parameter search space the algorithm considers and does not refer to any experimental temperature alterations. Each independent experiment was fitted separately. For fit analysis, 25 estimations with 0.5 permutations per type were used. Uncertainty estimates were calculated as the averages of the parameter obtained from each individual experiment. Fitting and fit analysis was performed with a modified version of IQMTools (Schmidt and Jirstrand, 2006) (https://iqmtools.intiquan.com/). All other software parameters not mentioned here were kept at their default values.

The used one-site receptor binding model assumes three possible interactions: the interaction between the receptor (R) and the fluorescence ligand (L), the receptor and the competitive unlabeled ligand (C) and non-specific binding sites from the receptor preparation (NBV) and fluorescent ligand. The corresponding reactions can be described by the following chemical equations:

$$R+L\overset{k_{{\;}_{+1}}}{\underset{k_{-1}}{⇄}}RL$$

$$R+C\overset{k_{{\;}_{+2}}}{\underset{k_{-2}}{⇄}}RC$$

$$NBV+L\overset{k_{{\;}_{+3}}}{\underset{k_{-3}}{⇄}}NBVL$$

Where *k*_*+x*_ *, k*_*-x*_ and *K*_*x*_ respectively correspond to the *k*_*on*_ *, k*_*off*_ and *K*_*d*_ or *K*_*i*_ of this reaction. The free parameters in the model were *k*_*on*_ (CELT-419 receptor binding), *K*_*d*_ (CELT-419 receptor binding), *k*_*on*_ (competitor receptor binding), *K*_*i*_(competitor receptor binding), *k*_*on*_ (CELT-419_non-specific binding), *FA* (Free CELT-419), *FA* (Receptor bound CELT-419), *FA* (non-specifically bound CELT-419), *R*_*stock*_ corresponding to the stock concentration of D_3_R in BBV-s and NBV_stock_.

For theoretical calculation of the apparent Log(IC_50_) predicted by the globally fitted model (Theoretical Log(IC_50_)), the model predictions of FA values at all used competitive ligand concentration points were predicted for each simulation time-point and subsequently fitted with the built-in three-parameter sigmoidal model (“log (inhibitor) vs. response”) in GraphPad Prism 5.04 (GraphPad Software, San Diego, United States).

To fit the Log(IC_50_) change in time from the competition binding experiments, a single-phase exponential decay equation with a constant corresponding to the equilibrium state was used. The model is described by the equation:

$${\mathrm{Log}\left({IC}_{50}\right)}_{t}=\left({\mathrm{Log}\left({IC}_{50}\right)}_{t=0}-{\mathrm{Log}\left({IC}_{50}\right)}_{equilibrium}\right)*e^{-kt}+{\mathrm{Log}\left({IC}_{50}\right)}_{equilibrium}$$ (3)

Where *t* is time, *k* is the exponential decay kinetic parameter, Log(*IC*_*50*_)_*t*_ is the predicted Log(*IC*_*50*_) at time point *t*, Log(*IC*_*50*_)_*t=0*_ is the apparent Log(*IC*_*50*_) at time point 0, and Log(*IC*_*50*_)_*equilibrium*_ is the predicted Log(*IC*_*50*_) at *t* = ∞.

To determine the assay suitability for HTS applications, Z’ values were calculated according to the formula (Zhang et al., 1999):

$$Z^{\prime }=1-\frac{3\left(\sigma_{positive\;control}+\sigma_{negative\;control}\right)}{\left|\mu_{positive\;control}-\mu_{negative\;control}\right|}$$ (4)

Where σ is the standard deviation of either the positive or the negative control respectively and µ is the arithmetic mean of the positive or the negative control respectively. Z’ value of over 0.5 is considered to be an assay suitable for HTS.

All the values calculated are weighted averages and the uncertainties given are weighted standard deviations of three independent experiments if not stated otherwise. Since the uncertainties obtained from global systems biology fit analysis were low, all the global analysis results are just means and the uncertainty is the standard error of the mean.

The octanol-water partition coefficient LogP for compounds was determined computationally using InstantJChem (ChemAxon) software.

## 3 Results and discussion

### 3.1 Initial characterization of CELT-419 in radioligand binding assay

To validate that the fluorescent ligand CELT-419 binds to the D_3_ receptor with sufficient affinity for FA assays and retains subtype selectivity of the pharmacophore, the radioligand binding method was used. This also allows the selection of better initial conditions for fluorescence assay design and optimization. The IC_50_ ± SEM value for CELT-419 binding to D_3_ receptor was determined to be 30.1 ± 1.7 nM corresponding to apparent K_i_ ± SEM = 15.7 ± 0.9 nM according to the Cheng-Prusoff model (Cheng and Prusoff, 1973). However, CELT-419 binds also to the D_2_ receptor in a similar affinity range (IC_50_ ± SEM = 78 ± 6 nM; K_i_ ± SEM = 44 ± 3 nM), while the affinity to D_4_ receptor is significantly worse (12% displacement at 10 µM). Although FA assay has been shown to work with a concentration of the probe close to 10 nM, in most cases, it is desirable to use subnanomolar affinity probes to achieve a good signal-to-noise ratio as well as reduce the concentrations of assay components for better HTS compatibility. At first glance, it may seem that CELT-419 does not have sufficient affinity for an ideal FA assay as the FA signal window between total and non-specific binding decreases. It must be considered that measured affinities between assays may vary due to differences in assay design and receptor source. The difference may be further amplified if Cheng-Prusoff model assumptions are not fulfilled, for example, if the equilibrium has not been reached. The latter situation can easily happen, as usually a single end-point time is chosen in radioligand screening, but the time required to reach equilibrium depends on the ligand binding kinetics, which is unknown *a priori*. Considering these factors, CELT-419 is a suitable reporter probe candidate for fluorescence anisotropy assay development.

### 3.2 Kinetic and equilibrium properties of CELT-419 binding to D_3_ receptors in BBVs

**FIGURE 1:**
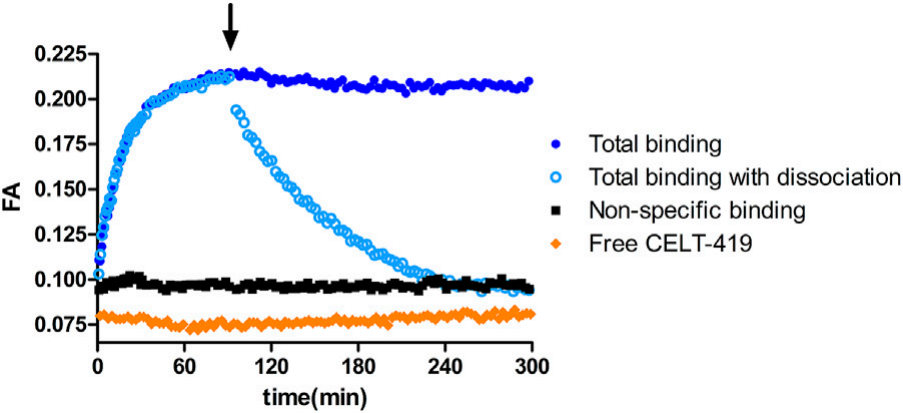
Time course of FA change caused by CELT-419 binding to D_3_ receptor on the BBV particles. The reaction was initiated by the addition of 1 μL D_3_ receptor displaying BBVs (C_D3R_ = 0.7 nM) to 0.5 nM CELT-419 in the absence (blue circles) or presence (black squares) of 50 μM Spiperone, or solution without BBVs (orange diamonds). After 90 min (indicated with an arrow), the measurement was paused, and dissociation was initiated by the addition of 333 μM Spiperone (open light blue circles). An equivalent volume of assay buffer was added to association controls (filled dark blue circles). A representative experiment of at least three independent experiments is shown.

Due to the homogeneous assay format, several luminescence-based assays allow online monitoring of ligand binding to receptors and dissociation of the receptor-ligand complex. In the case of the FA assay, the ligand must have a suitable fluorescent label to obtain good-quality results. The possibility to monitor the process kinetics is useful from multiple points of view – practically, it can be used to determine the time to reach equilibrium and, more fundamentally, to determine the kinetic parameters of the ligand binding. Taking the readout before reaching the equilibrium state can negatively affect the assay quality, as the affinities of competitive unlabeled ligands can be both over and underestimated depending on the situation, but FA assay allows to avoid this problem (Link et al., 2017). To study D_3_ receptors using FA assays, CELT-419 was designed based on a high-affinity pharmacophore and Cy3B label. Using the FA method, CELT-419 binding affinity and kinetics were determined using budded baculovirus particles (BBV) that display the D_3_ receptors on their membrane. Figure 1 shows that CELT-419 binds reversibly to the D_3_ receptor, and there is a large difference between total and non-specific binding resulting in a high signal-to-noise-ratio and stable signal over several hours. Good stability makes CELT-419 suitable for equilibrium state measurements. The FA of the free ligand is somewhat lower than the FA in the presence of BBVs and a high concentration of unlabeled ligand, indicating a low but measurable level of non-specific binding to BBVs. The model obtained by global analysis of kinetic data suggests that 8 ± 4% of CELT-419 is non-specifically bound.

**FIGURE 2:**
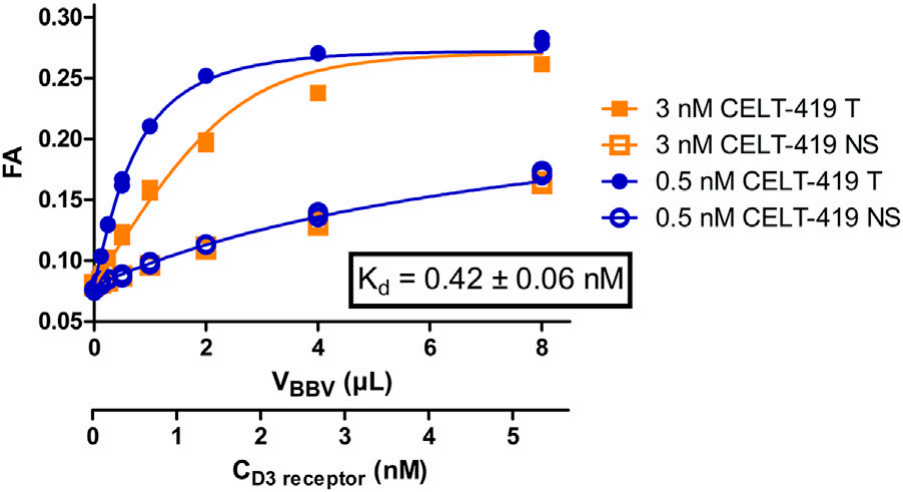
Binding curves of CELT-419 binding to D_3_ receptors in BBVs. FA of 3 nM (orange squares) or 0.5 nM (blue circles) CELT-419 were measured after 2 h incubation with different amounts of D_3_ receptor displaying BBVs. Non-specific binding (NS, open symbols) was determined in the presence and total binding (T, filled symbols) in absence of 50 μM Spiperone. The displayed fit, the concentration of D_3_ receptor binding sites and the K_d_ values were calculated *post hoc* from the results of these experiments using the model described in (Veiksina et al., 2014). K_d_ ± SEM is the weighted average of three independent experiments performed in duplicates. Representative experiment performed in duplicates is shown, with both duplicates displayed.

Saturation binding experiments with high-quality results (Figure 2; Supplementary Figure S2) enabled the calculation of the binding affinity constant K_d_ ± SEM of 0.42 ± 0.04 nM and receptor concentration in the BBV stock preparation R_stock_ ± SEM of 20 ± 4 nM. Unlike in the radioligand binding assay, FA assay requires ligand depletion and, therefore, second-order kinetic conditions. Since both the free ligand and free receptor concentrations change substantially during an experiment, it is necessary to know the R_stock_ to both design optimal experiments as well as to calculate K_d_, K_i_, k_on_ and k_off_ accurately. As FA assay enables monitoring ligand binding kinetics even during saturation binding experiments, it is alternatively possible to obtain all binding model parameters, including affinities, kinetic parameters and R_stock,_ in a single step. This can be achieved using global systems biology analysis of a single saturation binding experiment that includes both the association and dissociation phases (Supplementary Figure S1). This analysis revealed R_stock_kinetic_ ± SEM of 20 ± 5, k_on_ ± SEM of 4.2*10^–4^ ± 0.7*10^–4^ 1/(s*nM) and k_off_ ± SEM of 2.8*10^–4^ ± 1.2*10^–4^ 1/s resulting in K_d_kinetic_ ± SEM of 0.7 ± 0.3 nM which are in good correspondence with the K_d_ and R_stock_ values obtained from equilibrium analysis of the saturation binding experiment. The global analysis also revealed the FA ± SEM value of the free CELT-419 to be 0.081 ± 0.009 FA units (n = 6) which is in the expected range for Cy3B based fluorescence ligands. The relatively slow k_on_ of CELT-419 also shows that the initial radioligand screening was performed in a pre-equilibrium state, leading to apparently lower affinity in the radioligand binding assay.

### 3.3 FA competition binding assay with BBVs

**FIGURE 3:**
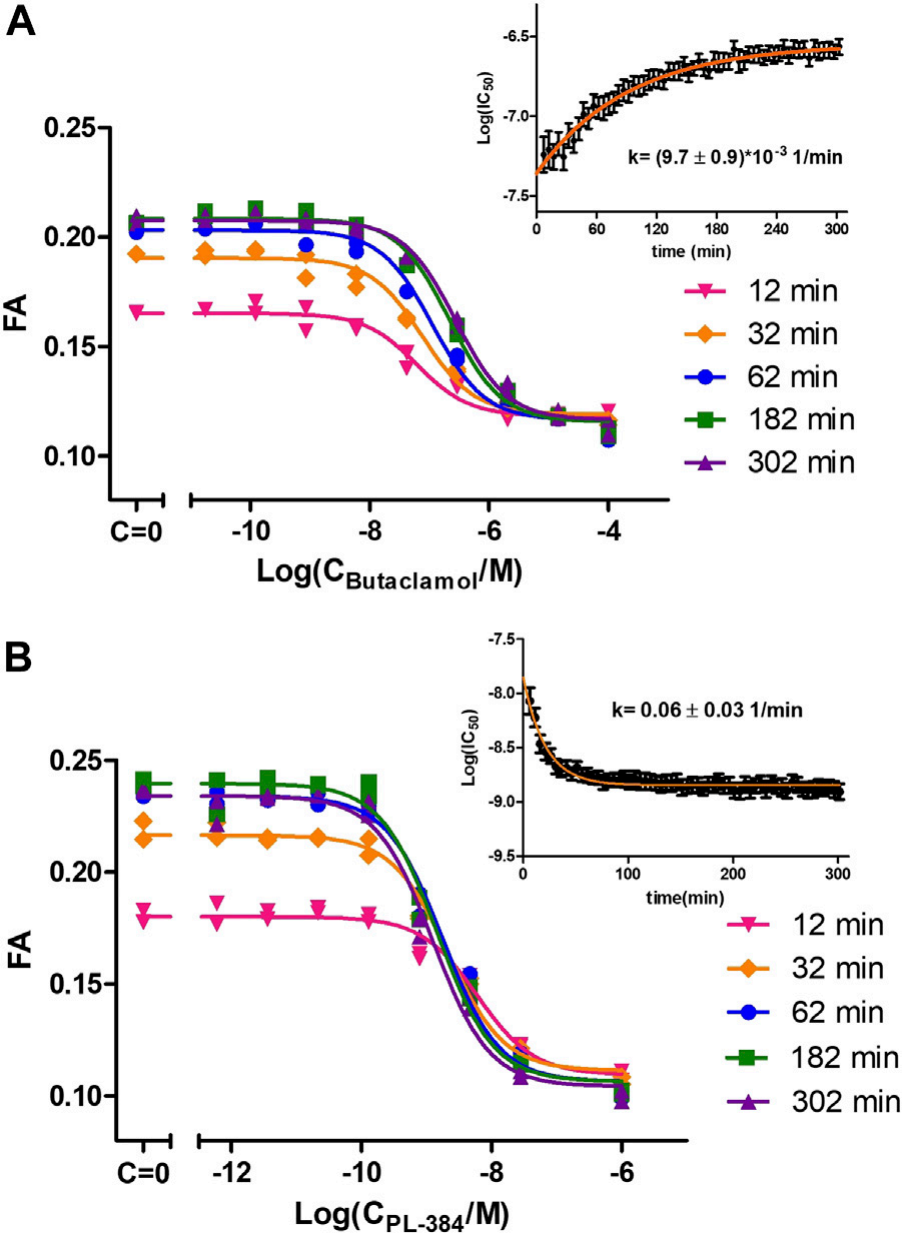
Displacement of CELT-419 by different concentrations of Butaclamol **(A)** and PL-384 **(B)** at different time points. The final concentration of CELT-419 was 0.5 nM and 1 μL BBV/well (C_D3R_ = 0.7 nM) was used. The insert shows the Log(IC_50_) ± SE change in time of corresponding displacement curves. Data from a single representative experiment performed in duplicate is shown. The Log(IC_50_) change in time was fitted with equation 3 and k given is the weighted average from 3 **(A)** or 4 **(B)** experiments with weighted SD.

The good affinity, high signal-to-noise ratio and stable FA signal during CELT-419 binding to D_3_ receptors of BBVs would make it suitable for measuring affinities of unlabeled ligands. For precise determination of unlabeled ligand affinities, it is important to consider the speed of reaching the binding equilibrium. In the case of conventional radioligand binding assay, it is quite a cumbersome task, which may require the measurement of several binding curves with different incubation times. With the FA assay, multiple samples can be monitored parallelly in real-time, thus allowing to observe how the displacement curve changes. This, in turn, allows monitoring IC_50_ value changes and stabilisation in time. The kinetic parameters of both the probe and the competitive ligand determine whether the Log(IC_50_) increases or decreases in time and how quickly the Log(IC_50_) values stabilize. Consequently, it is possible to derive conclusions about these kinetics parameters by observing Log(IC_50_) value change in time. For all tested classical dopaminergic ligands - Spiperone, Haloperidol, Dopamine, Apomorphine, 7-OH-DPAT and Butaclamol, the Log(IC_50_) values increased in time. This indicates these ligands bind to the D3 receptor faster than the probe CELT-419. The rate-limiting step to reach equilibrium is either CELT-419 association rate, the competitor´s dissociation rate, or both. As an example, the IC_50_ stabilisation for Butaclamol binding takes approximately 3 h after initiating the reaction (Figure 3A). However, an inverted behaviour of Log(IC_50_) values decreasing in time (Figure 3B) was present in the case of PL-384, a pharmacophore-linker moiety used for CELT-419 assembly. The time of stabilization of Log(IC_50_) values is considerably shorter for PL-384 having a half-life ± SEM of 8 ± 4 min (n = 4) (Figure 3B) compared to 71 ± 4 min for the Butaclamol competition curve (Figure 3A). Usually, faster stabilization time indicates smaller differences between the binding kinetics of the probe and the competitive ligand. Such kinetic information is essential for experimental design and interpretation of results. For example, the monitoring duration can potentially be dynamically shortened or extended, and more accurate affinity values could be obtained by IC_50_ or K_i_ extrapolation in time. In this case, the Log(IC_50_) stabilisation time course revealed that for any used competitive ligand using the 3 h time-point for Log(IC_50_) collection did not lead to substantial differences compared to later time-points and the resulting uncertainty was insignificant compared to variation between independent experiments.

**FIGURE 4:**
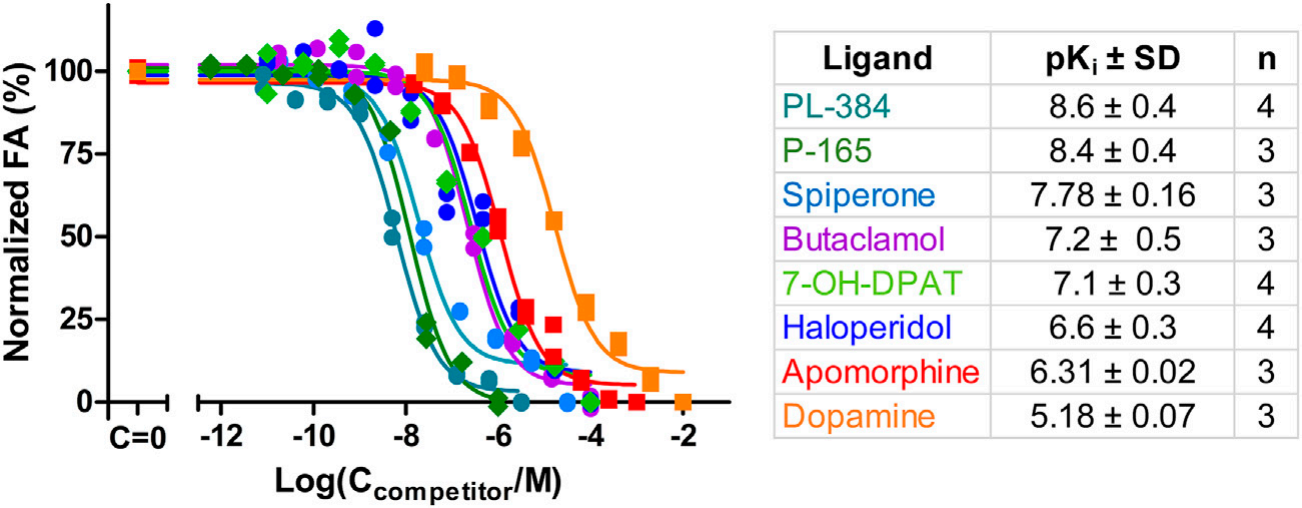
Displacement of CELT-419 binding to D_3_ receptor by different dopaminergic ligands. Change in FA level was measured after incubation of 0.5 nM CELT-419, 1 μL BBV/well (C_D3R_ = 0.7 nM), and different concentrations of corresponding dopaminergic ligands for 180 min. The FA values were normalized by taking the average FA value of C = 0 points as 100% and the average FA value of the highest used concentration points as 0%. Data of a representative experiment from at least three independent experiments, performed in duplicates, is shown with both replicates displayed. pK_i_ values are calculated as described in (Veiksina et al., 2014), and presented as mean ± SD of n independent experiments.

Ultimately, the most common use for FA assays is the indirect determination of interaction characteristics, most often the binding affinity, between a receptor and unlabeled ligands or allosteric modulators. To validate that the developed assay is suitable for measuring the K_i_ of novel ligands, competition binding measurements were carried out using CELT-419 as a reporter ligand for a set of established dopaminergic ligands (Figure 4). The assay shows a high signal level with ΔFA ±SD between total and non-specific points of 0.12 ± 0.02 (n = 26) anisotropy units. The estimated Z′ of the assay is 0.71, which is sufficient for high throughput screening standards, with an assay with Z’ above 0.5 considered excellent. The K_i_ values were calculated based on the IC_50_ values, receptor concentration and K_d_ of CELT-419 as described in (Veiksina et al., 2014), taking into account the ligand depletion effect during FA assay. Overall the determined affinities agree well with the affinities from previous studies.

**FIGURE 5:**
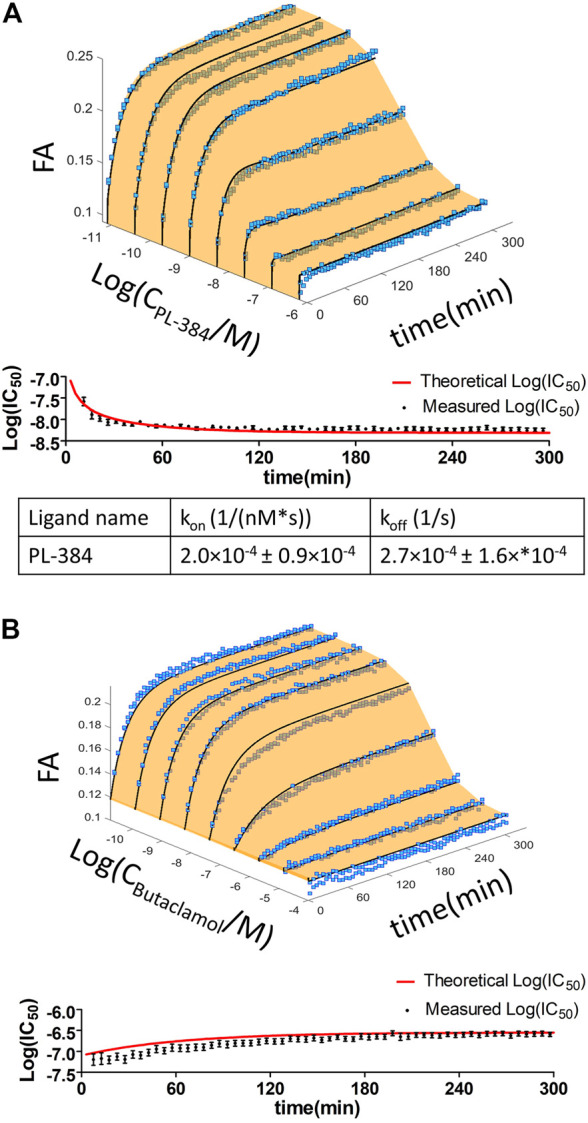
Time dependence of the displacement of 1 nM CELT-419 by increasing concentrations of PL-384 **(A)** and Butaclamol **(B)** obtained from FA competition binding experiments. Data of representative experiments performed in duplicate are shown. The light orange surface and black curves indicate the global fit of the single binding site model to the data from FA experiments. Log(IC_50_) ± SE values obtained from three-parameter sigmoidal dose response model of the raw FA measurement data (black circles) and of the global fitted data (red line), calculated at different incubation times, are shown in the lower panel of the respective graphs. k_on_ and k_off_ values shown are given as mean ± SEM of three experiments.

In addition to well-known dopaminergic ligands, the affinities of the P-165 (pharmacophore of CELT-419) and PL-384 (corresponding to the pharmacophore-linker moiety of CELT-419), synthesized and provided by Celtarys, were also determined to establish if and which effects the linker and fluorescent labelling have on the chemical and receptor binding properties of the ligand. The measured affinities of PL-384 and P-165 did not show any substantial differences from the affinity of CELT-419 (Figure 4), indicating that neither the linker nor the fluorescent label have noteworthy detrimental nor beneficial effects on the binding affinities of these compounds. Due to the relatively slow kinetics of PL-384 compared to CELT-419 (Figure 3B), it was possible to determine the kinetic parameters of PL-384 binding to D_3_ receptors from competition binding experiment data by global systems biology analysis (Figure 5). The model validity is supported by the correct prediction of the direction and kinetics of Log(IC_50_) values in time for different ligands. The k_on_ ± SEM and k_off_ ± SEM were determined to be 2.0 × 10^–4^ ± 0.9×10^–4^ 1/(nM*s) and 2.7 × 10^–4^ ± 1.6×*10^–4^ 1/s, respectively. Interestingly, the k_off_ values of PL-384 and CELT-419 are very similar, while k_off_ of P-165 is large enough that it cannot be accurately determined with this experimental setup. This suggests that adding a linker to the pharmacophore substantially slows down both association and dissociation kinetics, but further addition of the fluorescent label to the structure does not additionally affect the receptor-ligand complex dissociation kinetics, but the k_on_ of CELT-419 is larger than k_on_ of PL-384, which may be connected with higher hydrophobicity of PL-384 compared to CELT-419. Altogether, these kinetic aspects hint that the linker design and linking strategy may provide options for fine-tuning the fluorescent ligand kinetic properties. Whether this conclusion applies only to this pharmacophore and receptor combination or applies more generally to linker design remains unknown. Furthermore, the theoretically calculated water-octanol partition coefficients of P-165, PL-384 and CELT-419 were 6.14, 2.07 and 2.47, respectively, showing increased hydrophobicity introduced by conjugation with a linker can be reversed by subsequent fluorescent labelling. Higher hydrophilicity also increases ligand solubility and thus may reduce the risk of some common sample preparation and measurement problems, such as non-specific binding to plastic labware or biomembranes and aggregation with other assay components.

For an assay to be reasonable for HTS applications, it is useful to optimize costs by assay miniaturization. One possibility for that is to use a 384-well plate format for FA assay, which has been successful on some previous occasions but has not received sufficient attention (Vaasa et al., 2009; Huwiler et al., 2010) in terms of achievable Z′ values for in GPCR ligand binding assays. The results indicate that this setup gives a high Z’ value, thus proving that the change does not deteriorate the assay, and there is even a perspective to use the 1536-well format. However, some practical aspects must be considered when using the 384-well plate format. Firstly, pipetting liquid into the small diameter wells can lead to the formation of air bubbles, which substantially interfere with the measurement. Moreover, during more extended kinetic measurements, the liquid volume in the well may decrease substantially due to evaporation, which affects concentrations and may also affect fluorescence intensity values. For the 3–5 h experiments performed in this study, the evaporation did not substantially affect the final results.

### 3.4 Quantitative live-cell microscopy with CELT-419

**FIGURE 6:**
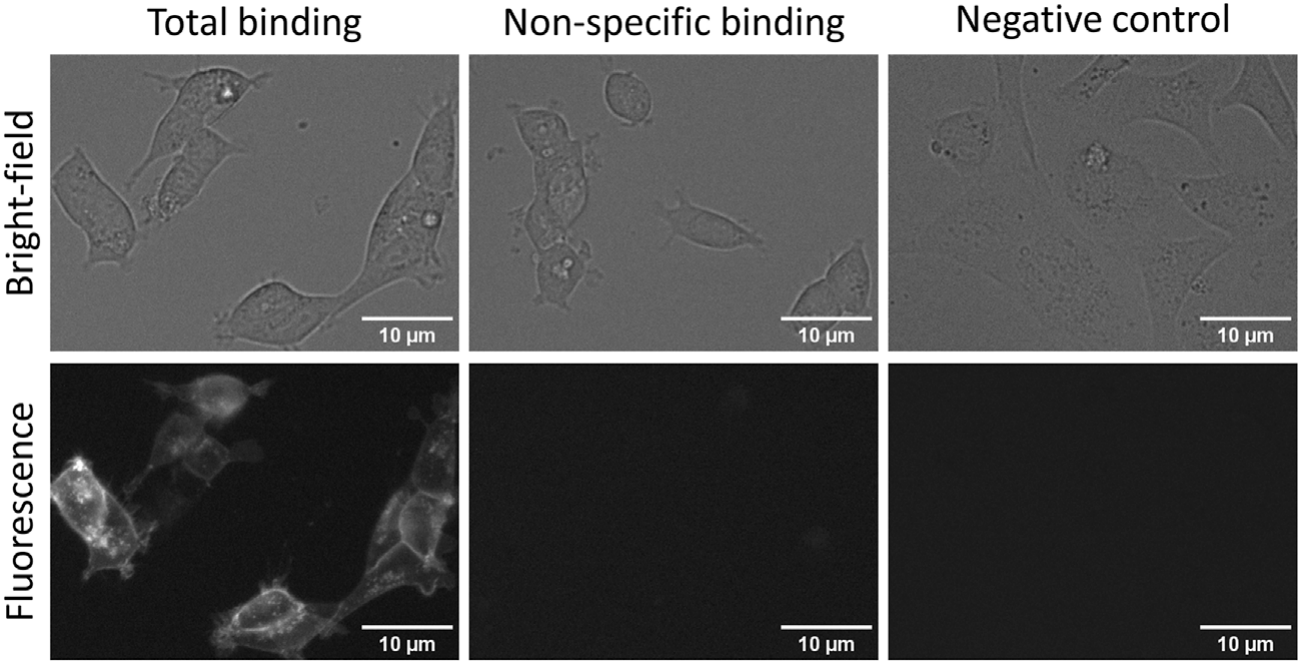
Fluorescence and bright-field images of total (left panels) and non-specific binding (central panels) of CELT-419 to live HEK293-D3R cells and to SKOV3 cells without D_3_ receptors (right panels). HEK293-D3R cells in DMEM medium with added 9% FBS and antibiotic antimycotic solution were incubated with 2 nM CELT-419 in the absence (total binding) or presence (non-specific binding) of 10 μM Spiperone for 2 h in 5% CO_2_ and at 37 °C. The number of seeded cells per well was 20,000. For negative control, SKOV3 cells without D_3_ receptors seeded at 30,000 cells per well were used with 2 nM CELT-419. The contrast of fluorescence and bright-field images was enhanced for presentation purposes only, the same lookup table was used for all images of the same imaging mode. The scale bar corresponds to 10 μm.

New robust fluorescent ligands and sophisticated microscopy systems have opened possibilities to perform more high-quality and automated measurements and, therefore, allows HTS-compatible quantitative ligand binding experiments in live cells. Although both FA and quantitative live-cell microscopy can be used for developing ligand binding assays, there are substantial differences in assay setups and receptor expression systems which must be considered. The obvious difference is the lack of receptor activation and downstream signalling in BBVs, which may further impact ligand binding. It would be a great advantage if the same fluorescent ligand could be used for cross-validation of multiple quantitative ligand binding methods, as has been done recently with M_4_ muscarinic receptors (Tahk et al., 2022). These two assays also open the possibility of choosing between throughput and spacial or temporal resolution and cost. A previously developed fluorescent ligand for D_3_ receptors NAPS-Cy3B worked well in a live-cell assay, but it was not directly compared with the FA assay (Allikalt et al., 2021). Therefore, CELT-419, which worked well in FA assays, was also tested with live HEK293-D3R cells. CELT-419 binding to D_3_ receptors in cells can be clearly visualized with fluorescence imaging. The signal is highly specific as no binding can be seen in the presence of 10 µM Spiperone nor in cells not expressing the D_3_ receptor (Figure 6). CELT-419 localizes mainly to the cell contour corresponding to receptor-ligand binding in cell membranes. These results indicate that CELT-419 is suitable for live-cell assay with automated microscopy. Furthermore, by employing deep convolutional networks for cell detection from bright-field images and Aparecium software for image and metadata pre-and post-processing, it is possible to develop fully quantitative ligand binding assays based on live-cell microscopy. This allows to vary fluorescence ligand concentration in a more classical saturation binding assay as well as to perform competition binding experiments using unlabeled ligands.

**FIGURE 7:**
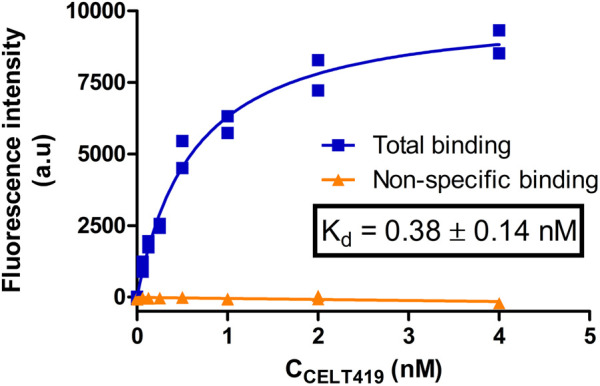
Saturation binding of CELT-419 binding to D_3_ receptors on live HEK293-D3R cells. The HEK293-D3R cells (20,000 cells per well) were incubated with CELT-419 (twofold serial dilutions up to 4 nM) for 4.3 h. Non-specific binding (orange triangles) was measured in the presence of 10 μM Spiperone. The background-corrected fluorescence intensities of cells were determined with the cell detection and image quantification software as described in Materials and methods and are presented as individual replicates from a representative experiment of three independent experiments performed in duplicates. Every point corresponds to the difference between the average pixel intensity of a cell and the average pixel intensity of the background. The average pixel intensities of each point were calculated from four images from different fields of view obtained from a single well. The calculated K_d_ value is given as mean ± SEM of three independent experiments.

As the next step, the cells were incubated with different concentrations of CELT-419 to determine its affinity for D_3_ receptors in a live cell system. For this, the previously proposed image quantification strategy was applied with the MembraneTools toolbox of Aparecium software (Tahk et al., 2022). The analysis uses a dedicated deep learning (DL) model U-Net for cell detection and subsequent quantification of cell intensity on the fluorescence images. As the intensity corresponds to relative amounts of bound fluorescence ligand, the binding affinity of CELT-419 to live HEK293-D3R cells can be determined*.* The K_d_ ± SEM of 0.38 ± 0.14 nM determined from the saturation binding curve (Figure 7; Supplementary Figure S3) is in good agreement with the affinity obtained from the FA assay (Figure 2). Furthermore, it is important that all described experiments were carried out in cell culture media with all the supplements, including FBS, to maintain cell growth conditions as close to normal as possible. In this case, a good signal-to-noise ratio could be achieved without the need to use DBPS or a specific live-cell imaging cell culture medium.

**FIGURE 8:**
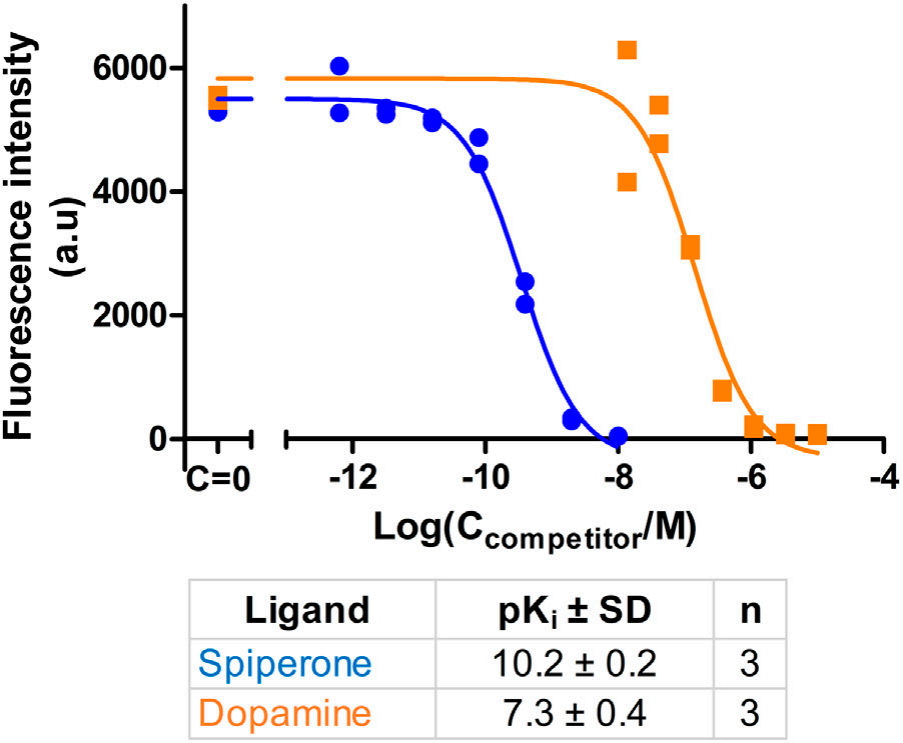
Inhibition of CELT-419 binding to live HEK-D3R cells by dopaminergic receptor ligands. The HEK293-D3R cells (40,000 cells/well) were incubated with 1 nM CELT-419 and different concentrations of Dopamine or Spiperone for 180 min as described in Materials and methods. The background-corrected fluorescence intensities of cells were determined with the cell detection and image quantification software as described in Materials and methods and are presented as individual replicates from a representative experiment of three independent experiments performed in duplicates. Every point corresponds to the difference between the average pixel intensity of a cell and the average pixel intensity of the background. The average pixel intensities of each point were calculated from four images from different fields of view obtained from a single well.

Also, competition experiments were carried out to demonstrate the versatility of this system (Figure 8; Supplementary Figure S4). Dopamine (agonist) as well as Spiperone (antagonist) caused concentration-dependent displacement of CELT-419 to live HEK293-D3R cells. The affinities of these ligands in live cells were higher than in FA assay, but this can be caused by difference in cell membrane composition and downstream signalling systems of the targets. As it is shown before that dopamine receptors ligands can achieve higher affinity if G-proteins are coupled to the receptor (Rinken et al., 2001).

### 3.5 Future perspectives

As can be seen from all the data from this study, CELT-419 is a high-affinity ligand with good kinetic properties, which showed similar results both in FA assay with BBVs and quantitative live-cell fluorescence microscopy. Therefore, both assays can be used for fundamental D_3_ receptor-ligand binding studies as well as for drug screening purposes. The use of CELT-419 as a fluorescent ligand is not limited by the methods used in this study. Several fluorescence ligands that performed well in either FA or live-cell assays have been confirmed to work well also, for example, in both live-cell and BBV-based TIRF microscopy assays, nanoBRET method and flow cytometry (Grätz et al., 2021; Laasfeld et al., 2021; Müller et al., 2022). In addition, this ligand may be suitable for some super-resolution microscopy methods such as photoactivated localization microscopy (PALM) or other single-molecule localization microscopy (SMLM) techniques (Dempsey et al., 2011; Schueder et al., 2019). However, the Cy3B label of CELT-419, which fits well for FA and live-cell microscopy measurements, is not the best choice for all assays. For example, some assays benefit from using a more red-shifted fluorescent label or a combination of multiple labels in a single study. For example, a Cy5-labelled fluorescent ligand with the same pharmacophore is available from Celtarys Research (CELT-241) which may be more suitable for tissue labelling and imagining due to lower autofluorescence. Altogether, the development of similar probes for other GPCRs and further development of measurement methods can increase the quality and quantity of both fundamental receptor research and high-throughput drug screening.

## Data Availability

The raw data supporting the conclusions of this article will be made available by the authors, without undue reservation.
