# Supplementary material for: Fluorescence based HTS-compatible ligand binding assays for dopamine D3 receptors in baculovirus preparations and live cells
Source: Front Mol Biosci. 2023 Mar 16;10:1119157. doi: 10.3389/fmolb.2023.1119157 (PMC10062709; doi:10.3389/fmolb.2023.1119157)
Supplement: Supplementary file 1 [file DataSheet1.docx]

Supplementary Material

Fluorescence based HTS compatible ligand binding assays for dopamine D_3_ receptors in baculovirus preparations and live cells

Maris-Johanna Tahk^1†^, Tõnis Laasfeld^1,2†^, Elo Meriste^1^, Jose Brea^3^, Maria I. Loza^3^, Maria Majellaro^4,5^, Marialessandra Contino^6^, Eddy Sotelo^5^, Ago Rinken^1*^

^†^ These authors contributed equally to this work and share first authorship.

*** Correspondence:**  [ago.rinken@ut.ee](mailto:ago.rinken@ut.ee)


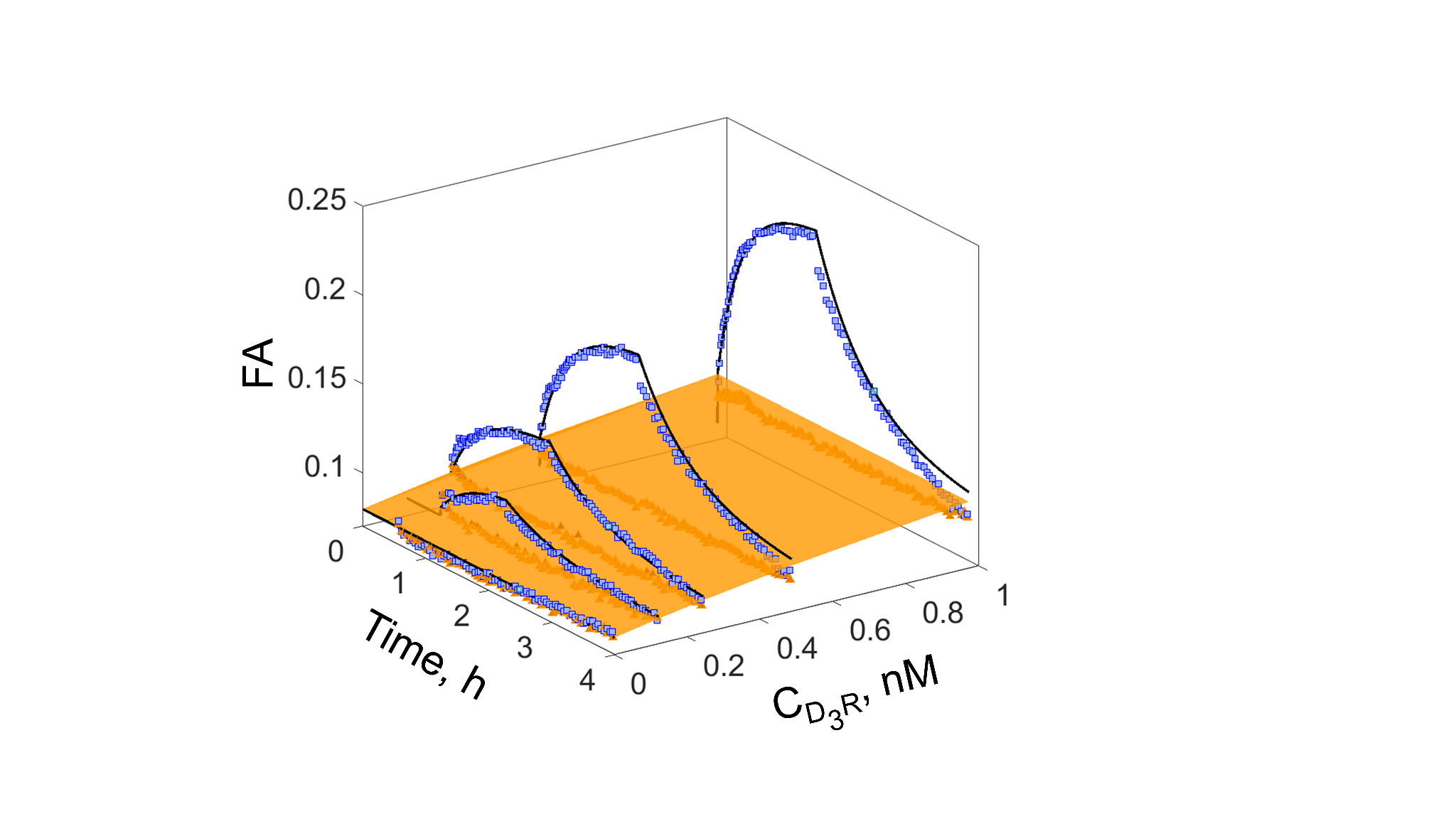


**Supplementary Figure 1.** Time course and corresponding global fit of FA change caused by CELT-419 binding to D3 receptor on the BBV particles. The reaction was initiated by the addition of a variable volume of D3 receptor displaying BBVs (1 µl BBV corresponding to c_D3R_ = 0.67 nM) to 0.5 nM CELT-419 in the absence (blue squares) or presence (orange triangles) of 50 μM Spiperone. After 120 min the measurement was paused, and dissociation was initiated by adding 333 μM Spiperone.

**
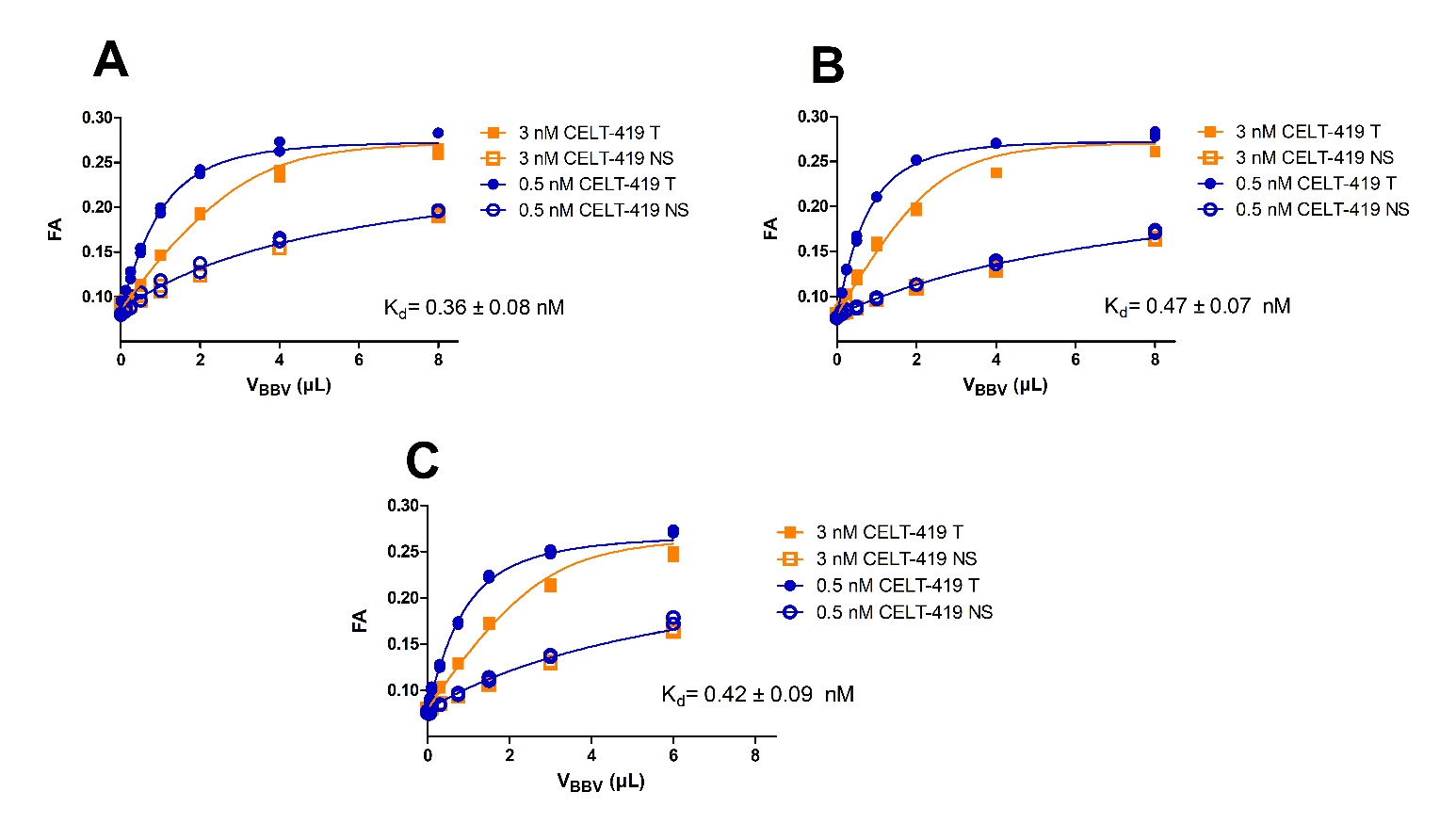
**

**Supplementary Figure 2.** Binding curves of CELT-419 binding to D_3_ receptors in BBVs. FA of 3 nM (orange squares) or 0.5 nM (blue circles) CELT-419 were measured after 2 h incubation with different amounts of D_3_ receptor displaying BBVs. Non-specific binding (open symbols) was determined in the presence of 50 μM Spiperone. The concentration of D_3_ receptor binding sites and the K_d_ were calculated post hoc from the results of these experiments using the model described in (Veiksina et al., 2014). Each of the panels (A, B and C) corresponds to a single independent experiment. Data shown in the figures are from a single experiment with both duplicates shown.


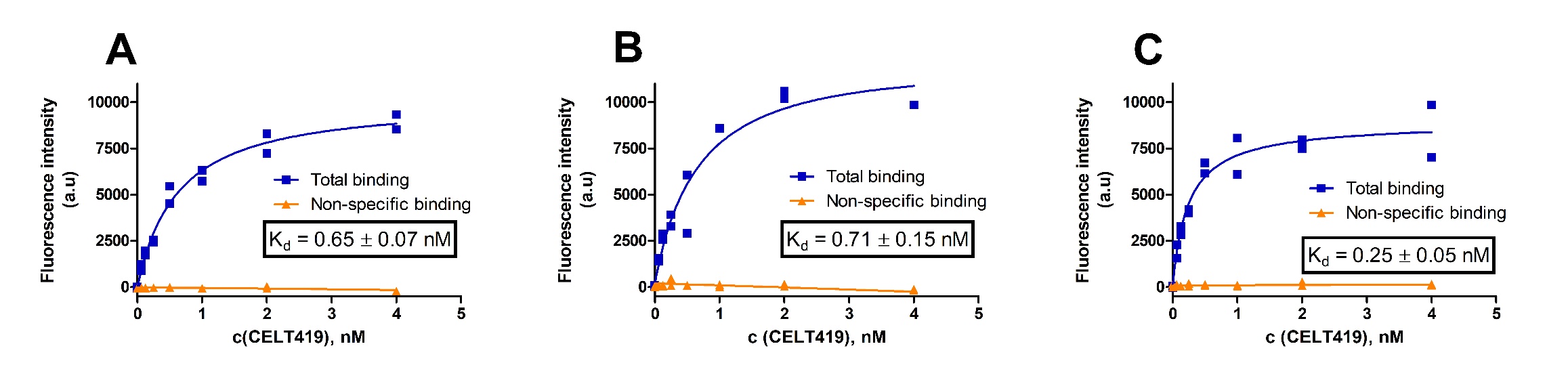

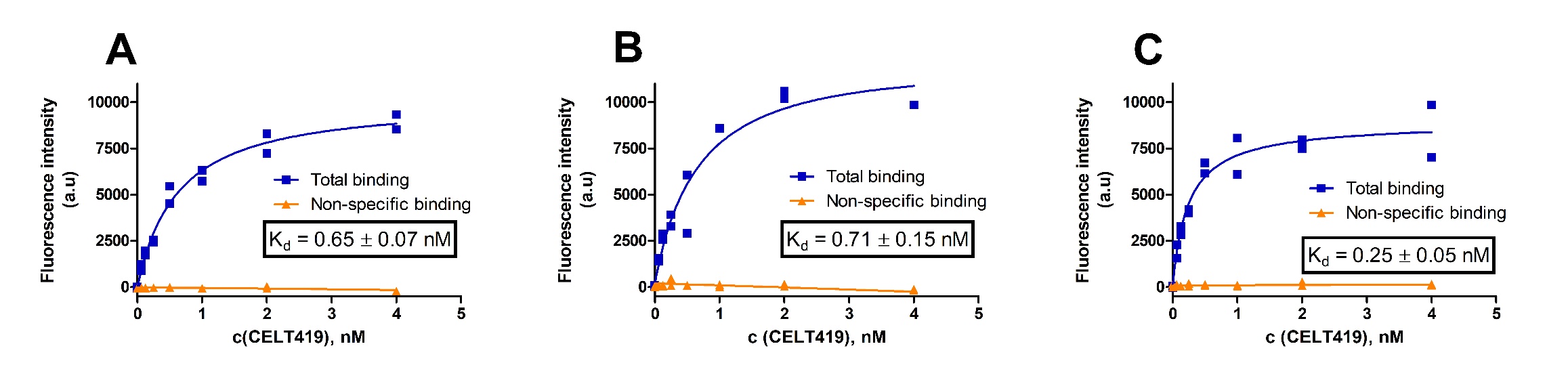


**Supplementary Figure 3.** Saturation binding of CELT-419 binding to D_3_ receptors on live HEK293-D3R cells. The HEK293-D3R cells (20 000 cells per well) were incubated with CELT-419 (twofold serial dilutions up to 4 nM) for 4.3 h. Non-specific binding (orange triangles) was measured in the presence of 10 μM Spiperone. The background-corrected fluorescence intensities of cells were determined with the cell detection and image quantification software as described in Material and methods and are presented as individual replicates from a representative experiment of three independent experiments performed in duplicates. Every point corresponds to the difference between the average pixel intensity of a cell and the average pixel intensity of the background. The average pixel intensities are calculated from four images from different fields of view obtained from a single well. Each of the panels (A, B and C) corresponds to a single independent experiment. Data shown in the figures are from a single experiment with both duplicates shown.


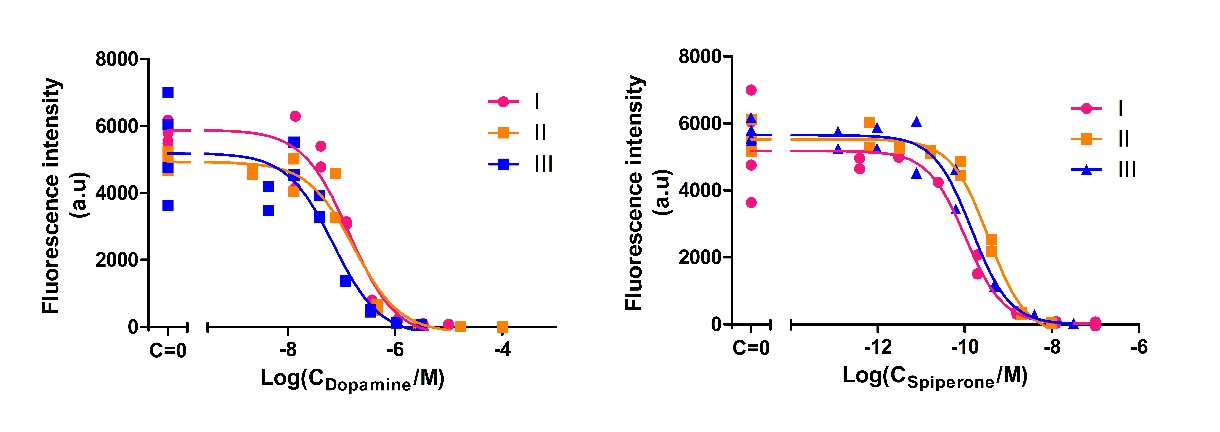


**Supplementary Figure 4.** Inhibition of CELT-419 binding to live HEK-D3R cells by dopaminergic receptor ligands. The HEK293-D3R cells (30 000 cells/well) were incubated with 1 nM CELT-419 and different concentrations of Dopamine or Spiperone for 180 min as described in Materials and Methods. The background-corrected fluorescence intensities of cells were determined with the cell detection and image quantification software as described in Material and methods and are presented as individual replicates. Each of the panels (A, B and C) corresponds to a single independent experiment. Every point corresponds to the difference between the average pixel intensity of a cell and the average pixel intensity of the background. The average pixel intensities are calculated from four images from different fields of view obtained from a single well.
